# Development and Pathology of the Equine Mammary Gland

**DOI:** 10.1007/s10911-020-09471-2

**Published:** 2020-12-05

**Authors:** Katherine Hughes

**Affiliations:** Department of Veterinary Medicine, University of Cambridge, Cambridge, UK

**Keywords:** Horse, Mammary gland, Mastitis, Milk, Pathology, Tumour

## Abstract

An understanding of the anatomy, histology, and development of the equine mammary gland underpins study of the pathology of diseases including galactorrhoea, agalactia, mastitis, and mammary tumour development. This review examines the prenatal development of the equine mammary gland and the striking degree to which the tissue undergoes postnatal development associated with the reproductive cycle. The gland is characterised by epithelial structures arranged in terminal duct lobular units, similar to those of the human breast, supported by distinct zones of intra- and interlobular collagenous stroma. Mastitis and mammary carcinomas are two of the most frequently described equine mammary pathologies and have an overlap in associated clinical signs. Mastitis is most frequently associated with bacterial aetiologies, particularly *Streptococcus* spp., and knowledge of the process of post-lactational regression can be applied to preventative husbandry strategies. Equine mammary tumours are rare and carry a poor prognosis in many cases. Recent studies have used mammosphere assays to reveal novel insights into the identification and potential behaviour of mammary stem/progenitor cell populations. These suggest that mammospheres derived from equine cells have different growth dynamics compared to those from other species. In parallel with studying the equine mammary gland in order to advance knowledge of equine mammary disease at the interface of basic and clinical science, there is a need to better understand equine lactational biology. This is driven in part by the recognition of the potential value of horse and donkey milk for human consumption, particularly donkey milk in children with ‘Cow Milk Protein Allergy’.

## Introduction

The mammary gland, or udder as it may be referred to in ungulates, is a fascinating organ that, compared to many other organs, is subject to an unusually high level of postnatal development during puberty and the reproductive cycle [1]. Whilst the equine mammary gland is relatively less frequently affected by disease than the ruminant udder by mastitis, and the mammary gland of companion animal carnivores by neoplasia, both mastitis [2] and mammary tumours [3] do occur in horses. In addition, the equine mammary gland has interesting developmental features, such as the striking similarity of the equine mammary gland to the human breast. Recent studies have revealed novel insights into the identification and behaviour of equine mammary stem/progenitor cell (MaS/PC) populations that have potentially profound implications for the understanding of mammary tumourigenesis [4]. Furthermore, understanding of specific facets of the mammary postnatal developmental cycle, particularly involution, underpins husbandry strategies aimed at reducing the incidence of mastitis in mares. However, comprehensive reviews of equine mammary development, and pathology of the equine mammary gland, are currently sparse. Thus, this review will provide an exploration of the development, and gross and molecular pathology of the equine mammary gland, that together underpin clinical understanding of equine mammary disease pathogenesis. Where relevant, comparisons will be drawn with humans and other species of veterinary or experimental interest.

## The Equine Mammary Gland in Health

### Gross Anatomy of the Mammary Gland

**Fig. 1:**
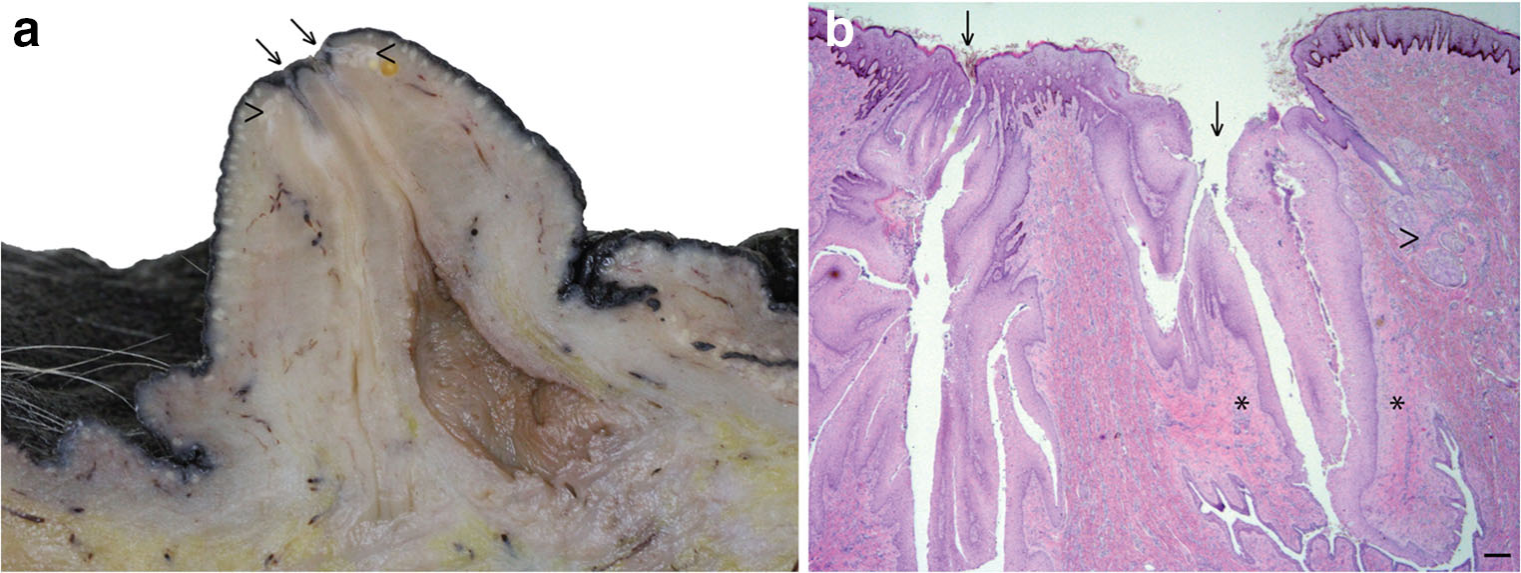
**The equine mammary gland is characterised by formation of two mammolobular-pilo-sebaceous units (MPSU) per teat.** Para-sagittal section through the distal mammary gland and teat of a mare (**a**) and corresponding histological section (**b**) demonstrating the two openings of the ductal systems (arrows) and corresponding sebaceous glands (arrowheads). b. Only one sebaceous gland unit (arrowhead) is visible in the histological section. Note the sparsely cellular connective tissue surrounding the teat canal (*). Haematoxylin and eosin stain. Scale bar = 200 μm

The equine udder comprises one pair of mammae each with a teat. Each mamma is usually drained by two independent mammary ductal trees, although three may rarely occur [5]. Thus each teat typically has two orifices through which the main ducts discharge (Fig. 1) [6]. This is in contrast to the cow which has two pairs of inguinal mammae, each drained by one ductal system. Like the horse, sheep and goats have only one pair of inguinal mammae, although unlike the horse, but similar to the cow, these only have one ductal system per mamma [7]. Cats and dogs have four and five pairs of mammae respectively, with multiple ductal trees per mamma [8, 9]. Rabbits similarly have four or five pairs of mammae, each with 6–7 ductal systems [10].

In contrast to male ruminants that have rudimentary mammary structures, the majority of male horses lack teats [11]. However, some male donkeys have teats on their sheath [12] and this is also the case in some mules (A. Mclean, personal communication) [11].

### Prenatal Mammary Development

The equine anatomical arrangement of one pair of inguinal mammae, each having two ductal trees [13], arises during foetal development. The embryonic mammary gland has ectodermal (epithelial) and mesenchymal (stromal) cellular compartments. Across species, initial mammary development is considered to be characterised by the formation of ventral milk lines, exhibiting an anterior-to-posterior alignment, and composed of multi-layered ectoderm [14] although the formation of the milk line is somewhat controversial in the horse [6, 11]. Complex interplay between ectodermal and mesenchymal compartments results in thickenings of ectoderm called mammary placodes developing at expected locations along the milk line [14] and these are suggested to be present at 7.9 cm foetal crown-rump length in the horse [6]. The cells of the mammary placode then differentiate into the mammary bulb at approximately 8 cm equine foetal crown-rump length [6].

At approximately 9.5 cm foetal crown-rump length, two tendrils of cells arise from the mammary bulb and grow into the underlying mesenchyme. These are called primary sprouts. Ensuing foetal development results in secondary sprouts developing from the primary sprouts, and it is the descending secondary sprouts that give rise to the teat cistern and ductal tree. A lateral secondary sprout forms a pilosebaceous sprout that develops into primary, and on occasion secondary, hair follicles together with a sebaceous gland. The associated hair(s) exit the teat adjacent to the mammary duct that also originated from the primary sprout. Thus development of the equine mammary gland is characterised by formation of two mammolobular-pilo-sebaceous units (MPSU) per teat, each MPSU comprising a galactophorous duct, a mammary hair, and a sebaceous gland [6]. Intriguingly, the immature domestic cat also exhibits MPSU at approximately one week after birth, but the pilosebaceous component regresses by approximately 3 months of age [6]. By contrast, in the horse the MPSU persist postnatally and can be clearly observed in adult horses (Fig. 1).

### Equine Mammary Gland Structural Organisation and Histology

**Fig. 2:**
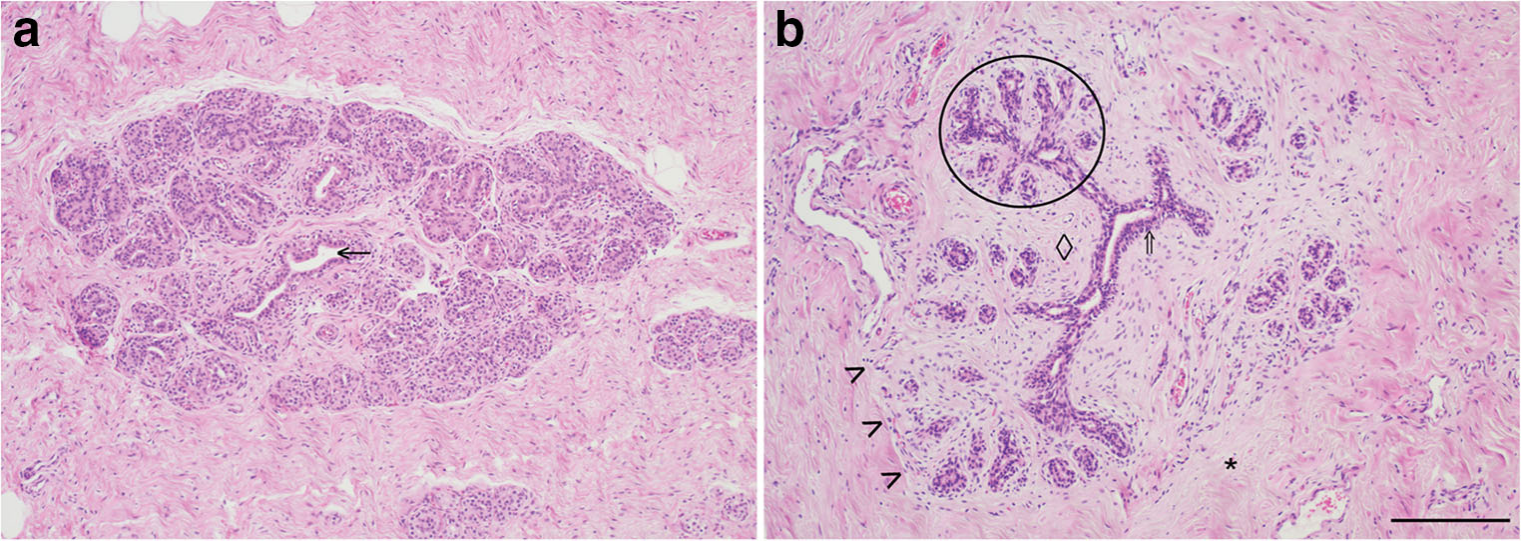
**The equine mammary gland is characterised by terminal duct lobular units (TDLUs) supported by collagenous stroma. a**. Quiescent mammary tissue from a 28 year-old thoroughbred cross mare of unknown reproductive history. The lobular arrangement of the mammary acini and ducts (arrow) is clearly apparent. **b**. Quiescent mammary tissue from a 12 year-old warmblood of unknown reproductive history. The bilaminar arrangement of epithelial cells within the ducts is distinct (double arrow). A TDLU is encircled. Note the more cellular intralobular stroma (diamond) supporting TDLUs, and the more loose and sparsely cellular interlobular stroma (*) which surrounds lobules. The border between the two types of stroma is particularly well-demarcated in this image (arrowheads). Haematoxylin and eosin stain. Scale bar = 200 μm

The equine mammary gland is characterised by collagenous stroma in which the epithelial structures are arranged in terminal duct lobular units (TDLUs) similar to the TDLUs of the human breast [15]. The ruminant mammary gland is also organised into TDLUs [16] that are from time to time described as analogous terminal duct units [17, 18]. A TDLU comprises a group, or lobule, of blind-ending mammary acini and both intralobular and extralobular portions of the subtending terminal duct, which together comprise the functional unit of the mammary gland (Fig. 2). In the human breast TDLUs drain into the intralobular terminal ducts, and subsequently the extralobular terminal ducts, subsegmental ducts, and segmental ducts, which eventually converge to form the primary mammary ducts [15]. Murine mammary ducts have bulb-shaped structures called terminal end buds that are key in coordination of subsequent duct growth and branching [19, 20].

**Fig. 3:**
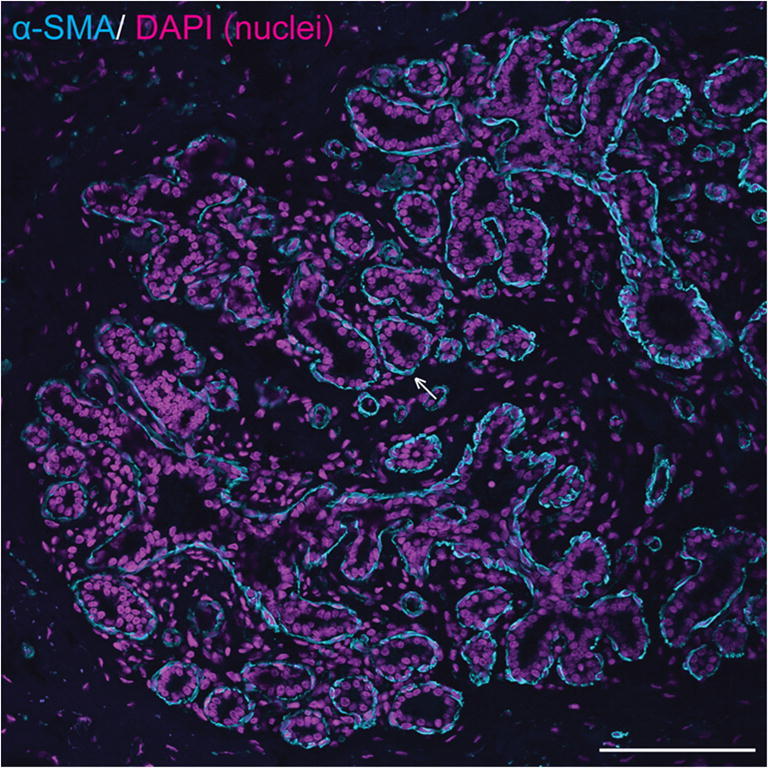
**Equine mammary epithelial cells exhibit a bilaminar arrangement, with expression of alpha smooth muscle actin (α-SMA) in the outer myoepithelial layer**. Immunofluorescence staining for α-SMA (cyan) and DNA (DAPI; magenta) in equine mammary tissue from a 16 year-old thoroughbred mare. Arrow indicates myoepithelial cell expressing α-SMA. Scale bar = 116 μm. Image is representative of three biological repeats from different mares

Mammary ducts are bilayered, with basal and luminal layers of mammary epithelial cells visible [10, 21, 22] (Fig. 2b; Fig. 3). Unlike the basal epithelial cells of stratified squamous epithelia, mammary basal epithelial cells exhibit characteristics common to smooth muscle cells, including expression of alpha smooth muscle actin (α-SMA) [23–25], and hence may be referred to as ‘myoepithelial’ cells to emphasise their contractile phenotype [26, 27]. As would be anticipated, equine mammary myoepithelial cells also exhibit α-SMA expression (Fig. 3) [28].

**Fig. 4:**
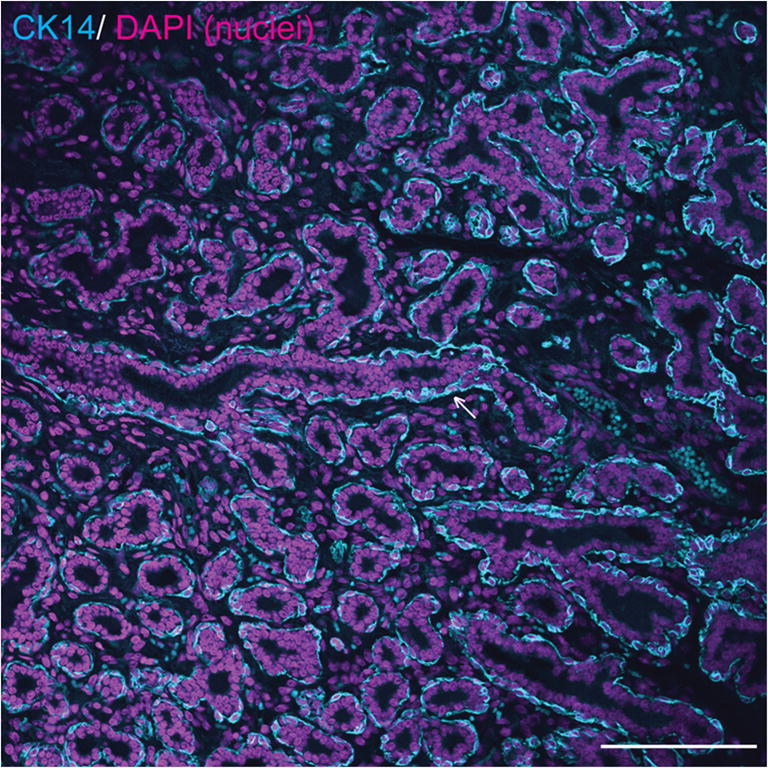
**Equine mammary myoepithelial cells express cytokeratin 14 (CK14).** Immunofluorescence staining for CK14 (cyan) and DNA (DAPI; magenta) in equine mammary tissue. Arrow indicates ductal basal epithelial cell expressing CK14. Scale bar = 116 μm. Image is representative of three biological repeats from different mares

Basal and luminal mammary epithelial cells may also be distinguished by their expression of intermediate filaments, although this may vary between species and between locations within the mammary gland, for example expression of specific cytokeratins (CKs) may be different between epithelial cells in large ducts and those within a TDLU [20, 26]. In humans, the high molecular weight cytokeratins CK5 and CK14, which can form heterodimers, are expressed in interlobular duct basal myoepithelial cells but are predominantly expressed in luminal epithelial cells in TDLUs [20, 26]. By contrast, in the adult mouse mammary gland, CK5 and CK14 are considered to be expressed solely by myoepithelial cells [20, 29]. In the adult rabbit mammary gland during pregnancy and lactation, CK14 is expressed predominantly in the epithelial basal compartment of ducts and sinus-like dilatations, but with occasional positive luminal epithelial cells [10]. Based on the currently available evidence, in the adult horse CK14 is similarly expressed predominantly in mammary basal epithelial cells [3, 30] (Fig. 4).

CK7 and CK18 are expressed in luminal mammary epithelial cells in the human breast [31, 32]. In the adult horse, CK8/18 is also expressed in the luminal mammary epithelial cells, although expression is somewhat weak with a commercially available antibody [30]. Equine mammary luminal epithelial cells similarly express CK8 [28].

In the vast majority of investigations of equine mammary biology and pathology, antibodies raised against epitopes in species other than the horse will be utilised. The above discussion of variable expression of intermediate filaments between different species highlights the need for particularly stringent evaluation of such experiments, and the requirement for appropriate control tissue [30]. An ideal positive control tissue will also include areas where the antigen of interest is not expressed (negative internal control) [33]. For example, the basal aspect of stratified epithelium can be used as a positive control for CK14 [26] together with the myoepithelial cells of nearby dermal apocrine glands, whilst the apocrine gland luminal epithelia are a negative internal control for CK14. Where possible it is desirable to perform confirmatory western blotting to demonstrate detection of an appropriately sized protein in the species of interest. The variability of mammary intermediate filament expression between different species also represents a major challenge in the delineation of MaS/PCs in species such as the horse, a subject that will be examined in more detail later.

The stroma of the equine mammary gland is arranged into more densely packed collagenous and cellular intralobular stroma in which TDLUs are embedded, and looser and more sparsely cellular interlobular stroma which surrounds lobules (Fig. 2b). Interestingly, a similar pattern is seen in ruminants, with intralobular stroma and comparable ‘near stroma’, and interlobular stroma and equivalent ‘far stroma’, recognised [7, 16, 17, 34, 35]. The equine and ruminant mammary stroma thus shows similarities to the human breast [15]. By contrast, the stroma of the mouse mammary gland is adipocyte-rich and quite different from that of the horse, human and ruminant [20].

As already described, each equine teat exhibits two MPSU. Similar to ruminants [36], the equine mammary teat canal is surrounded by sparsely cellular connective tissue and smooth muscle fibres (Fig. 1).

### Equine Postnatal Mammary Development

As in other species, the mammary gland exhibits a dramatic level of tightly-regulated postnatal growth and development, particularly associated with physiological events such as puberty, pregnancy, lactation, and post-lactational regression. Such growth and development is coordinated by a number of factors including steroid hormones [37] and the STAT family of transcription factors [38]. Unfortunately, compared to experimental rodents and production ruminants, there is little specific data available regarding equine mammary postnatal development. Given the presence of significant species differences in mammary gland biology, extrapolation of results across species, particularly from rodent models, necessitates significant caution and is not always appropriate [39]. However, some general comments may be made from comparison with other species.

To better describe the postnatal developmental fluctuations in the human breast, some authors classify breast lobules according to their degree of complexity. Type 1 lobules are the simplest and comprise a terminal duct surrounded by a grouping of alveolar buds. Alveolar buds are considered to be more developed than a terminal end bud but less complex than the terminal structures of the mature mammary gland known as acini. Type 2 and 3 lobules are distinguished by an increased lobular area and more numerous alveolar buds that resemble ductules. In humans, lobule formation occurs 1–2 years after puberty, and the breasts of nulliparous women are predominantly composed of type 1 lobules, with smaller numbers of type 2 and 3 lobules [40]. Similar lobules can be observed in other species that exhibit TDLUs, including horses (Fig. 2). Interestingly, proportions of type 1, 2, and 3 lobules present in the porcine mammary gland have been demonstrated to be similar to the proportions in the human breast [41]. During pregnancy in women, a growth phase heralds proliferation of distal elements of the ductal tree, with ductules developing into acini, a signature that marks conversion of type 3 lobules into type 4 lobules [22].

These structural changes within the breast or mammary gland are underpinned by hormonal influences and through experiments carried out in ovariectomized pigs, oestrogen has been demonstrated to promote progression between type 1, 2 and 3 lobules [41]. Experiments in ovariectomized calves may similarly have relevance to the understanding of hormonal influences on postnatal mammary gland development. In this species it has been demonstrated that ovariectomy causes a reduction in mammary parenchymal mass [42]. Furthermore, prepubertal ovariectomy abrogates progesterone receptor expression in mammary epithelial cells and reduces the intensity of oestrogen receptor expression. Tissue from ovariectomized calves exhibits reduced abundance of Ki67, a nuclear protein expressed in cells that are actively cycling, both in the mammary epithelial cells and in the stroma [43].

As is evident from the above discussion, the relative paucity of specifically equine-focussed information regarding the molecular biology of mammary postnatal development highlights this as an area where future studies might be usefully directed.

### Equine Lactation and Milk Biology

High concentrations of oestrogens and progestogens occur during pregnancy [13]. The final week of pregnancy heralds a marked rise in plasma prolactin concentration. Prolactin levels remain elevated to a variable degree in early lactation, before an eventual reduction to basal levels by 1–2 months post foaling [44]. Prolactin binding to its receptor activates the transcription factor Signal Transducer and Activator of Transcription 5 (STAT5) which initiates expression of milk protein genes in mammary epithelial cells [1]. Further information regarding hormonal regulation of lactation has been previously collated [13].

#### Milk Let Down

When a mother suckles her offspring, the maternally derived neuropeptide oxytocin binds to its cognate receptor expressed on mammary basal (myoepithelial) cells stimulating intracellular calcium signalling that results in contraction of the myoepithelial cells and milk expulsion [45]. This process is known as ‘milk let down’. Anticipation of nursing, stimulation of the udder by the foal, and nursing itself, can all precipitate release of oxytocin from the posterior pituitary [13, 46].

#### Colostrum

Colostrum is the first form of milk produced by the mammae. On the first day of lactation, equine colostrum comprises approximately 25% total solids and 2.85–2.93% fat. Concentration of vitamins A, D_3_, K_3_ and C is 1.4–2.6 times higher than in normal milk [47]. Total protein concentration in the region of 16% has been documented [48]. Analysis of colostrum, and milk samples taken during the first week of lactation, has revealed that the oligosaccharide profile of equine milk has shared features with human, bovine, porcine, and caprine milk, but also distinct differences [49, 50].

Mean colostral concentrations of IgG, IgM, and IgA have been reported as 8911.9 ± 6282.2 mg/dl, 957 ± 1088.1 mg/dl, and 122.9 ± 77.3 mg/dl, respectively [51]. Density of colostrum has been used as a surrogate read-out of IgG levels and, for equine colostrum, has been suggested to be 1060 g/l [52]. In the cow, refractive index has also been shown to correlate with IgG concentration [53] and it is asserted that good quality equine colostrum should have a 23% brix value [52]. As might be anticipated, weight loss in the pregnant mare may impact colostrum quality and milk yield [54]. It is possible that the quality of colostrum produced by older mares may be reduced [55].

#### Milk

Relative to the milk of most domestic species, total proteins, fat, inorganic salts, and energy are low in equine milk, but mare’s milk is rich in lactose [13, 56] and lactose concentration may slowly increase as lactation progresses [57]. Caseins comprise approximately 80% of bovine total milk proteins, but by contrast equine milk contains less casein (∼55% of total protein) and more whey proteins [56].

Interestingly, compared to the milk of ruminants, horse and donkey milk are considered to be more similar in composition to human milk [56]. Donkey milk is proposed as a less allergenic alternative to cow’s milk for children with ‘Cow Milk Protein Allergy’ [58, 59]. This is in part due to the aforementioned lower levels of αs1-casein compared to cow’s milk [58]. Furthermore, for human infants, taurine is as an essential metabolite and whilst equine milk has ten times less taurine than human milk, it has notably more taurine than bovine milk [56].

A number of factors, including genetic and environmental considerations, and stage of lactation, likely influence the composition of mare’s milk. Mare age may be a contributory factor, and interestingly, milk composition may also influenced by breed, although this is a somewhat controversial suggestion (reviewed in [56]).

#### Cytology of Colostrum and Milk

Cytological examination of equine colostrum or milk may be undertaken. Smears of colostrum collected within 12 h of parturition are characterised by a granular or homogeneous protein background with fragmented nuclear debris and red/purple spheres. Clear lipid vacuoles and epithelial cells with a large vacuole (signet or secretory cells) may be detected in some cases. Smears of equine milk again have a proteinaceous background and are either acellular or may contain scarce neutrophils [60, 61].

### Post-Lactational Mammary Regression or Involution

The term post-lactational mammary regression, or involution, describes the dramatic changes occurring within the mammary gland at the end of lactation [38]. Across species, in both experimental and natural settings, involution is initiated following weaning that may be abrupt or gradual [62]. Understanding the biological process of mammary post-lactational regression is important as it underpins husbandry measures taken around the time of weaning to reduce mastitis incidence in mares.

The process of involution has been most extensively characterised following abrupt, or forced, weaning in rodents. From such studies it has been demonstrated that involution is a bipartite process. The first, reversible phase is driven by local factors, presumably stimulated by milk accretion [63] resulting in increased intraluminal pressure [64]. These signals result in extensive STAT3-regulated cell death [65, 66], occurring by a lysosomal-mediated cell death pathway [67–69]. Further cell death also occurs in the second irreversible phase of involution. This phase exhibits hallmarks of profound tissue remodelling and immune cell infiltration, that together have caused it to be likened to a healing wound [70–72]. During this irreversible remodelling large numbers of leucocytes, particularly macrophages [24, 25, 73], are present. The macrophages exhibit a STAT3-dependent immunomodulatory phenotype [74] and participate in involution-associated neo-lymphangiogenesis [75]. Cytology affords a valuable window into mammary involution in the mare and correlates with these observations from mice in that vacuolated macrophages predominate in secretions collected during early involution. By contrast, secretions from later involution are characterised by cells that are small and dark, with minimal cytoplasm. These may be shrunken epithelial cells or lymphocytes [61].

Potentially pertinent to the likely progression of involution in mares, cell death also occurs during involution induced by natural weaning in experimental rodents although the cell death dynamics may differ, with slower onset of cell death in the context of natural weaning [76]. Also relevant to some mares, that will be pregnant during involution, is the recognition that involution in dairy cows often proceeds with a ‘parallel pregnancy signature’ [7, 35]. In mice, concurrent pregnancy reduces the magnitude of cell death initiated following abrupt weaning [77] although cell death occurs earlier in mice that are subjected to natural weaning whilst pregnant [76]. Overall, it has been observed that mammary involution in remated mice is quite different from the process observed in non-pregnant rodents [76] and similarly it is likely that the dry period in dairy cows represents not only a phase of cell death but also a period of epithelial cell renewal [78, 79]. Although dairy cows have been selectively bred for high milk yields, it would seem possible, or even likely, that the mammary gland of mares exposed to the hormonal and cytokine milieu of concurrent pregnancy and involution may also exhibit dual features of cell death and cell proliferation and have a distinct time course and gene expression patterns compared to involution in non-pregnant mares.

### Senile Mammary Involution or Lobular Involution

In addition to post-lactational involution resulting from abrupt or gradual weaning, a form of mammary involution that is not associated directly with the cessation of lactation occurs at the end of the reproductive life of an animal [80]. In humans this senile mammary involution may be associated with a reduction in the number of type 3 mammary lobules, potentially reflecting the regression of type 3 lobules to a type 2 and 1 lobule phenotype [22].

### Equine Mammary Stem/Progenitor Cells

During embryonic development, the mammary gland is generated from multipotent mammary stem cells, and a subpopulation of MaS/PCs persist postnatally and are responsible for the dramatic postnatal mammary gland development associated with events such as puberty and pregnancy. The role of MaS/PCs and whether they contribute to only the luminal or basal epithelial lineages (unipotency) [81] or both lineages (bipotent MaS/PCs) remains controversial even in the mouse. This concept has been recently reviewed [82, 83], and will not be discussed further here.

Equine MaS/PCs have attracted interest due to the low incidence of mammary neoplasia in this species. They also likely contribute to maintenance of epithelial cell numbers during lactation [84]. However, there are a number of challenges surrounding working with MaS/PCs outside of the rodent and human arenas, and one particular issue is that panels of markers used to identify MaS/PCs in one species may not necessarily be applied with confidence to a different species [85]. One methodology that is not reliant on marker identification is to digest mammary tissue and to generate floating cell colonies called mammospheres enriched in MaS/PCs [84, 86]. Using this technique, it has been shown that equine mammospheres have different growth dynamics compared to canine mammospheres, and in particular, the mammosphere formation efficiency exceeds that of the canine mammospheres and is maintained for a much increased number of passages [87]. Intriguingly, microvesicles appear to contribute to the self-renewal signals through Wnt signalling pathways [87]. When compared to their canine counterparts, equine MaS/PCs also respond differently to agents causing DNA damage, activating the intrinsic and extrinsic apoptotic pathways. This may be a property contributing to the lower mammary cancer incidence in this species [4].

## Pathology of Diseases Affecting the Equine Mammary Gland

### Galactorrhoea

The term ‘galactorrhoea’ refers to inappropriate secretion of milk or a milk-like product from the mammary gland and encompasses both precocious secretion during pregnancy and inappropriate secretion in the absence of a prior lactation event [13]. These two different manifestations of galactorrhoea will be considered in turn below.

#### Precocious Lactation

In pregnant mares, precocious mammary development and lactation have been suggested to be often associated with imminent abortion, placentitis, or separation of the placenta [13] although spontaneous resolution of premature lactation in a mare carrying a viable foal and mummified foetus has been documented [88]. A report has also described premature udder development and lactation in a mare found to be carrying a dead foetus and receiving weekly administration of long-acting progesterone [89]. Notably, if mares develop galactorrhoea prior to foaling, there may be impaired passive transfer of immunoglobulins to the foal via colostrum due to loss of immunoglobulins in the earlier secretions [13, 88].

#### Inappropriate Lactation in Non-pregnant Mares

Mammary development and attendant inappropriate lactation may be observed in neonatal foals, where, as in newborns of other animal species and babies, the secretory product is colloquially referred to as ‘witch’s milk’ [11]. This phenomenon has been attributed to exposure to the mare’s lactogenic hormones [13]. Interestingly, as alluded to above, secretion of ‘witch’s milk’ has been detected in a notable proportion of human babies, where the pathogenesis of secretion is thought to be similar to that in foals [90]. Adult mares may also occasionally be presented with galactorrhoea [91] with potential causes including a lack of dopaminergic suppression of prolactin secretion, leading to increased prolactin levels, occurring secondary to equine pituitary pars intermedia dysfunction (equine Cushing’s disease), and exposure to oestrogens. Many cases, however, remain idiopathic [91–93].

### Agalactia

Agalactia describes the state where a female that should be producing colostrum or milk has an absence of lactation [13]. The condition has been described in a variety of species including dogs and cats, where the literature distinguishes two forms of agalactia according to underlying pathogenesis. Temporary agalactia is recognised in situations such as parturition in primiparous females or those undergoing premature caesarean sections and is symptomatic of a lack of synchrony between mammary development and parturition. By contrast, true agalactia reflects persistent lack of lactation with a variety of potential underlying aetiologies which may pertain either directly to the patient or to the patient’s environment [94]. In addition to small domestic carnivores, agalactia has been described in diverse species including pigs [95, 96], rabbits [97, 98], and horses [99, 100].

The clinical impact of agalactia in the horse can be profound as it may result in failure of passive transfer and insufficient nutrition for the foal [13]. Authors of equine texts distinguish between failure of milk let-down in maiden or stressed mares [13], which might be termed temporary agalactia by the above small animal definitions, and causes of true agalactia. In horses, mycotoxicosis is a notable cause of true agalactia and is typically associated with the consumption of the ergot alkaloids produced by the fungi *Claviceps purpurea* and *Neotyphodium coenophialum* (synonym *Acremonium coenophialum*). The latter infects *Festuca arundinacea* (synonym *Festuca elatior*). Hypogalactia or agalactia occurs secondary to decreased prolactin secretion and retarded mammary gland development [99–101]. The cause of decreased prolactin secretion is the dopamine agonist- and serotonin antagonist-effects of the alkaloids [13]. *Streptococcus equi* infection has also been implicated as a cause of true agalactia [102]. In other cases of equine agalactia, it is not possible to establish the underlying cause of the syndrome [103].

### Mastitis

#### Clinical Presentation and Diagnosis

Mastitis is an uncommon condition in horses [104] that most frequently occurs during lactation, or during post-lactational regression associated with weaning, and is therefore most commonly seen in the summer or autumn months [105]. In addition, mastitis may occur in association with milk build up relating to illness or loss of a foal, and may also be diagnosed in pregnant mares [106], non-pregnant dry mares [107], young fillies [108, 109] and neonates [110]. The relatively low frequency of mastitis in mares is a phenomenon that has attracted interest and discussion (Table 1) [104, 108].

**Table 1:** Factors implicated in the low frequency of mastitis in mares

Factor	Reference
Anatomical considerations	
Size of udder (smaller than cows)	[104]
Udder more concealed	[108] [104]
Teats less prone to trauma and infection	[104] [119]
Physiological considerations	
Small capacity of udder leading to frequent emptying	[104]
Short lactation period	[108]
Hyopthetical immunological or endocrine influences	[104]
Husbandry factors	
Small capacity of udder leading to frequent emptying in animals that are milked	[104]
Husbandry of mare and foal	[108]
Under-reporting due to poor identification of subclinical and low grade cases	[108]

Mares from a range of breeds have been reported as affected by mastitis. These include thoroughbreds, standardbreds, quarter horses and ponies [107, 109]. It is likely that the reported breeds are, to an undetermined degree, a reflection of prevalence of breeds in the caseloads of institutions where authors have studied mastitis.

The majority of mares are likely to present with unilateral disease, and in some cases only one ductal tree within a mamma may be affected [2, 109]. In one study of 28 cases, 50% of patients exhibited temperature exceeding 38 °C [2]. Swelling of the mammary gland, local heat, pain, purulent discharge and ventral oedema are common clinical signs. More severe cases may be accompanied by systemic signs of illness including depression, anorexia, and even ipsilateral hind limb lameness [2, 109]. Importantly, there is considerable overlap between many of these local and systemic clinical signs and those of mammary neoplasia (see next section).

Diagnosis is based on a combination of clinical signs, microbial culture and cytological evaluation. The cytological picture is usually one of myriad viable or degenerate neutrophils, necrotic material, and potentially other unidentifiable degenerate cells [60]. Importantly, bacteria are only detectable cytologically in approximately 30% of cases but conversely, the cytological picture may be very useful in diagnosing cases of mastitis where culture is negative [2] [60]. In addition, a minority of mares, likely those with systemic signs, may show neutrophilia and hyperfibrinogenaemia [2, 109].

#### Causes

Bacteria are the most commonly identified aetiological agents of equine mastitis. Whilst a range of gram positive and gram negative bacteria have been implicated, in one study in the USA *Streptococcus* spp. were the most frequently reported cause [2]. Cases of mastitis caused by *Corynebacterium* spp., *Streptococcus* spp., and *Staphylococcus* spp. may progress to abscess formation in some instances [5, 61]. Interestingly, it has recently been suggested that mammary abscesses may be more common mares from regions in which pigeon fever, caused by *Corynebacterium pseudotuberculosis*, is endemic [5]. Indeed, equine mammary abscesses from which *C. pseudotuberculosis* has been cultured have been previously reported [111]. Mammary infection with *Staphylococcus* spp. may elicit granulomatous inflammation, with a very distinctive pattern of response to the bacteria known as botryomycosis [93, 112].

In addition to bacterial causes of mastitis, fungal, parasitic, and toxic aetiologies have also been recorded. Fungal agents causing equine mastitis include *Coccidioides immitis* [113] and *Blastomyces dermatitidis* [114] whilst *Halicephalobus deletrix* and *Cephalobus* spp. are examples of parasitic aetiologies [115, 116]. In addition, there is a possibility that cutaneous habronemiasis [117] may affect the skin overling the mammary gland, or superficial portions of the gland itself. Avocado (*Persea americana*) is a potential mastitis-causing agent [118].

#### Concepts of Mammary Gland Biology that Underpin Prevention of Equine Mastitis

The main facets of mastitis prevention in horses focus dually on general husbandry measures and nutrition of mare and foal. In terms of husbandry, attentive udder monitoring and cleaning, and instigating husbandry measures that reduce the risk of acquisition of traumatic lesions, are both important. Implied in udder cleaning is adoption of measures to reduce the burden of flies and insects that may have a role in mastitis pathogenesis. Recognition of the importance of mammary acinar distension in the initiation of the involution process [63, 64] (see section on involution above) underpins advice to clients not to milk the mare after weaning [5].

There are two aspects of mastitis prevention specifically concerning nutrition. The first is the need to reduce mare dry matter intake at the point of drying-off to reduce milk production [5]. If the mare is fed excessively during the weaning phase, the gland may become inflamed and hardened, with the induration resolving gradually over time [119]. Providing creep feed for foals prior to drying off is also important in mastitis prevention [5]. Drying off is effectively synonymous with a forced involution in the experimental context. By providing generous creep before this point, a natural weaning process is fostered prior to forced involution and this is likely to reduce mastitis incidence.

### Mammary Tumours

#### Clinical Presentation and Diagnosis

Equine mammary tumours are rare [3] (reviewed in [120]), predominantly of epithelial origin, and are, in the majority of cases, malignant, although a mammary adenoma has also been documented [121]. The limited data available suggest that the prognosis is poor for mares diagnosed with this tumour type, with the potential for metastasis to regional lymph nodes and other organs [117, 122, 123].

Laterality, the dominance of one side over the other, has been interrogated in human breast cancer [124, 125]. In the case of equine mammary tumours, insufficient cases have been recorded to allow any conclusions to be drawn regarding laterality, but there is a necessity, therefore, to carefully record such information, so that data from multiple reports can be collated.

The postnatal mammary developmental cycle, incorporating dramatic waves of proliferation, cell death and remodelling as described earlier, fundamentally impacts susceptibility to mammary tumourigenesis in humans. A full-term birth at an early age and prolonged breast feeding both confer protective effects, whereas pregnancy and involution are periods associated with an increased risk of tumour development [126]. At least three pregnant mares with concurrent mammary neoplasia have been reported [127, 128] and recently a lactating mare that was presented with a mammary comedocarcinoma has been described [129]. Similar to the situation with laterality, no conclusions can currently be drawn regarding impact of mare reproductive history on mammary tumour development. However, with meticulous data recording and collaborative efforts, eventually sufficient data may exist to facilitate such investigations [30].

At the time of presentation to the clinician, equine mammary masses may be painful [130] or non-painful [131], and in some cases are accompanied by oedema ventrally [128, 132] or affecting the hind limbs [133]. In the case of animals with advanced metastatic disease at the point of clinical presentation, poor body condition or overt emaciation may be non-specific clinical signs [123, 128, 133]. Mares with pulmonary metastases may show signs of coughing or respiratory distress [130].

One key finding which emerges from the case reports and case series documenting this neoplastic entity is that mares with mammary tumours are frequently presented with clinical signs compatible with mastitis [3, 128] and distinction between mastitis and mammary neoplasia is an important facet of clinical assessment of mammary masses in the mare [30]. It has been suggested that misdiagnosis as mastitis is a notable cause of delay in achieving a diagnosis of an equine mammary tumour [134]. In this regard, it is important to note that cytology may be helpful in differentiating these two conditions if epithelial cells demonstrating unequivocal criteria of malignancy are aspirated [128, 135]. However, attendant inflammation may accompany mammary tumours and so an inflammatory cytological picture does not rule out underlying neoplasia [134]. Presence of ulceration has been postulated to be a factor that should increase clinical suspicion of neoplasia rather than mastitis [127, 128, 136]. Unfortunately, whilst the above assertions are potentially helpful diagnostic pointers, many are based on weak evidence and the above discussion again highlights that it is imperative to perform larger, multi-centre studies to accumulate sufficient cases to recognise diagnostically helpful trends. Ultimately, a core or excisional biopsy is frequently required for definitive diagnosis [134].

In a large number of published cases euthanasia is undertaken, potentially motivated by welfare or economic considerations, or on the basis of a likely poor prognosis. Therefore, the number of cases for which true survival data is available is strikingly small. Whilst very little can be definitively concluded regarding prognosis, a notable subset of published equine mammary tumours exhibited evidence of visceral metastatic spread at post mortem examination [3, 28, 123, 127, 130, 133, 136].

#### Histopathological and Molecular Analyses

One of the challenges for the histopathologist examining an equine mammary tumour is the current lack of a unifying diagnostic classification system. Given the spectrum of morphological variants encountered in the published literature, it is potentially feasible and advisable to currently follow the guidelines available for small companion animals [137]. Notably, a number of specific morphological variants described for feline and canine mammary tumours have been described in horses. For example, invasive micropapillary carcinoma [138] and ductal carcinoma [28] have both been documented.

Owing to the rarity of equine mammary carcinomas, very limited molecular analyses have been undertaken when compared to studies of mammary tumourigenesis in cats and dogs. Expression of intermediate filaments has been assessed in small numbers of equine mammary carcinomas and preliminarily indicates that carcinomas are heterogeneous, with documented examples of tumours expressing CK8 or CK18 (luminal epithelial markers), CK14 (basal marker), pan-cytokeratin, glial fibrillary acidic protein (GFAP) and vimentin, with varying degrees of intensity [3, 28, 139]. Positive staining for α-SMA has also been reported [139]. Thus equine mammary carcinomas may have the potential to exhibit luminal or basal phenotypes perhaps analogous to those seen in breast cancer and mammary carcinomas in other species of veterinary importance.

**Fig. 5:**
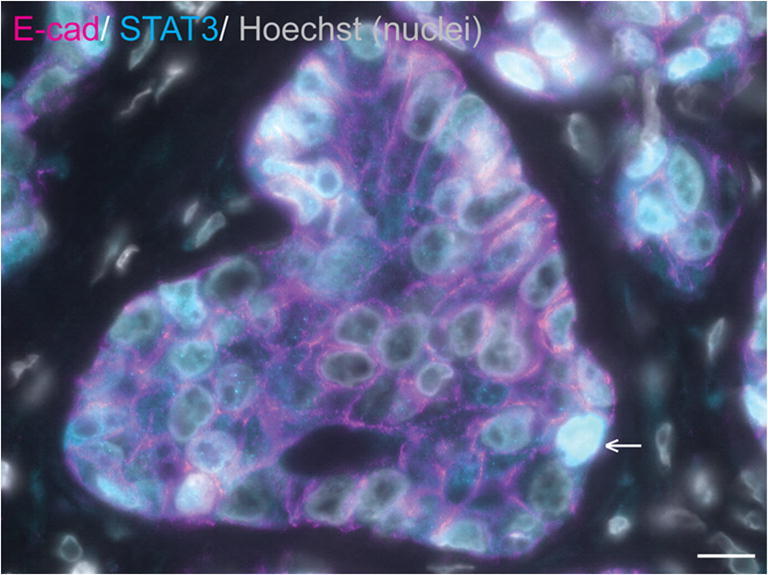
**STAT3 is expressed in a subset of equine mammary tumours.** Immunofluorescence staining for E-cadherin (E-cad; magenta), STAT3 (cyan) and DNA (Hoechst; grey) in an equine mammary carcinoma. Data from [3]. Arrow indicates a neoplastic cell exhibiting nuclear STAT3 expression. Scale bar = 10 μm

Similarly, a subset of equine mammary carcinomas exhibit oestrogen receptor alpha positivity by immunofluorescence, which has been suggested to correlate with weak or absent expression of vimentin [3]. Tumour oncogenes and tumour suppressor genes have also attracted interest in the study of equine mammary neoplasia. STAT3 is a known breast oncogene [140–142] that may bestow a direct survival advantage on breast cancer cells in addition to modulating the tumour microenvironment to facilitate neoplastic cell survival and/or infiltration and invasion. In one study, 3/7 equine mammary carcinomas exhibited nuclear STAT3 expression, implying transcriptional activation of STAT3 [3] (Fig. 5). By contrast to STAT3, p53 is a tumour suppressor gene for which inactivation through mutation confers resistance to apoptosis. Interestingly, in one mammary carcinoma mRNA levels of p53 were reduced compared to those observed in non-neoplastic equine mammary tissue [139]. These studies, although limited in both number and scope, illustrate the enormous potential for future interrogation of the molecular profile of equine mammary tumours.

#### Mammary Tumours of Non-mammary Origin

Occasional infiltration of the mammary gland by carcinomas not specifically of mammary origin may occur. For example, vulval, perineal, and mammary invasion by a large squamous cell carcinoma has been described in an 18-year-old Appaloosa mare [143]. Additionally, as might be expected, neoplasms of non-epithelial origin may also arise in the equine mammary gland. Recorded examples have included a malignant fibrous histiocytoma [144], lymphoma [145], and malignant melanoma [146]. Sarcoids may also theoretically arise in the region of the mammary gland [117].

## Conclusions and Future Perspectives

Whilst the equine mammary gland is not an organ commonly associated with disease, it is evident that pathology of this tissue, particularly mastitis and mammary neoplasia, frequently have profound effects on the welfare of mares and, in the case of mastitis, their foals. In addition, in some contexts, equine mammary disease has an economic, as well as a welfare impact. In the context of mammary tumours, the prognosis for affected mares may be bleak.

Due to the relatively low frequency of occurrence of many mammary diseases, much of the knowledge base in this field is built upon case reports and modest case series. Irrespective of an individual’s perspective on the value of veterinary case reports, such publications do have significant inherent limitations and lie at the base of the evidence-based medicine pyramid [147]. This review underlines the need for complementary larger multi-centre investigations that will facilitate application of evidence-based veterinary medicine to diseases of the equine mammary gland [148].

Underpinning clinical studies of mammary gland disease are those investigations which probe the biology of this fascinating tissue. Here, again, there is a relative paucity of equine-focussed literature. As has been stated earlier, extrapolation of data from other species is not without difficulty and risk of false assumptions. In addition, a lack of equine-specific reagents necessitates significant care when using antibodies and experimental reagents developed for use in human or murine subjects.

Recent publications have begun to elucidate the intriguing biology of equine MaS/PCs whilst also demonstrating the pressing need to better characterise the equine mammary stem cell hierarchy. Results from these publications, together with the relative rarity of equine mammary tumours, suggest that equine MaS/PCs may have particular functional properties meriting further investigation. Comparative mammary gland biology focussing on the horse may reveal new insights into tumourigenesis with relevance for humans and other species.

The use of donkey milk as an alternative milk for children with ‘Cow Milk Protein Allergy’ reinforces the importance of the study of equine lactational biology and underlines the need to focus on the biology of the equine mammary gland. Indeed, there is a growing scientific field concerned with optimising milk production in donkeys maintained for this purpose [149, 150].

Unanswered questions regarding the equine mammary gland in health and disease therefore abound, and this organ remains an exciting subject of study for basic scientists and veterinarians alike.

## Materials and Methods for Unpublished Experiments

Sections used for histology and immunofluorescence were obtained from tissues collected from cases examined post mortem by the anatomic pathology service of the author’s institution, that were surplus to diagnostic requirements. Consent for retention of tissues for teaching and research purposes was granted at the time of submission for post mortem examination. Mammary tissue was collected in 10% neutral-buffered formalin. Following fixation, tissues were processed, sectioned and staining with haematoylin and eosin following standard histological protocols. Immunofluorescence staining for α-SMA (ab124964, rabbit monoclonal [EPR5368] to α-SMA, dilution 1:2000) (Abcam, Discovery Drive, Cambridge Biomedical Campus, Cambridge) and CK14 (ab7800, mouse monoclonal [LL002] to CK14, dilution 1:200) (Abcam) was carried out manually on unstained sections cut from formalin fixed paraffin embedded tissue. De-paraffinisation and antigen retrieval were performed using Agilent Envision Flex Target Antigen Retrieval Solution High pH (Agilent Technologies LDA UK Limited, Life Sciences and Chemical Analysis Group, Lakeside, Cheadle Royal Business Park, Stockport, Cheshire) in an Agilent PT link pre-treatment module (Agilent Technologies LDA UK Limited) for 20 min at 90 °C. Standard protocols for immunofluorescence were followed [151].

## Data Availability

The data that support the figures in this manuscript are available from the corresponding author upon reasonable request.
